# Alexithymia Is Associated With a Multidomain, Multidimensional Failure of Interoception: Evidence From Novel Tests

**DOI:** 10.1037/xge0000366

**Published:** 2017-11-20

**Authors:** Jennifer Murphy, Caroline Catmur, Geoffrey Bird

**Affiliations:** 1 MRC Social, Genetic and Developmental Psychiatry Centre, Institute of Psychiatry, Psychology and Neuroscience, King’s College London; 2 Institute of Psychiatry, Psychology and Neuroscience, Department of Psychology, King’s College London; 3 MRC Social, Genetic and Developmental Psychiatry Centre, Institute of Psychiatry, Psychology and Neuroscience, King’s College London; Institute of Cognitive Neuroscience, University College London; Department of Experimental Psychology, University of Oxford

**Keywords:** alexithymia, interoception, p factor, autism spectrum disorder, anxiety

## Abstract

Interoception, the perception of the body’s internal state, contributes to numerous aspects of higher-order cognition. Several theories suggest a causal role for atypical interoception in specific psychiatric disorders, including a recent claim that atypical interoception represents a transdiagnostic impairment across disorders characterized by reduced perception of one’s own emotion (alexithymia). Such theories are supported predominantly by evidence from only one interoceptive domain (cardiac); however, evidence of domain-specific interoceptive ability highlights the need to assess interoception in noncardiac domains. Using novel interoceptive tasks, we demonstrate that individuals high in alexithymic traits show a reduced propensity to utilize interoceptive cues to gauge respiratory output (Experiment 1), reduced accuracy on tasks of muscular effort (Experiment 2), and taste sensitivity (Experiment 3), unrelated to any co-occurring autism, depression, or anxiety. Results suggest that alexithymia reflects a multidomain, multidimensional failure of interoception, which is consistent with theories suggesting that atypical interoception may underpin both symptom commonalities between psychiatric disorders and heterogeneity within disorders.

The study of interoception has undergone something of a resurgence in recent years. The term interoception refers to the perception of the body’s internal state (Craig, 2002). As such, hunger, thirst, respiratory, and cardiac signals are all interoceptive in nature. Whilst the term interoception was initially used exclusively to refer to visceral sensations (e.g., Fowler, 2003), contemporary definitions have expanded its use to refer to bodily signals that do not readily meet the criteria to be considered internal (e.g., sensual or affective touch, tickle, taste, and muscular exertion) but which are processed by common neural pathways (e.g., Craig, 2005; Wilson, Andrew, & Craig, 2002; Löken, Wessberg, Morrison, McGlone, & Olausson, 2009; Craig, 2002). Thus, more recent definitions of interoception include bodily information sent either via (a) small diameter (unmyelinated) C-fibers or (myelinated) Aδ-fibers, lamina I, the spinothalamic tract, and then on to the insula and anterior cingulate cortex—the spinal homeostatic pathway (Craig, 2002), or (b) cranial nerves (vagus and glossopharyngeal) to the nucleus of the solitary tract and on to the insula and anterior cingulate cortex—the cranial homeostatic pathway (Critchley & Harrison, 2013; see also Ceunen, Vlaeyen, & Van Diest, 2016; Murphy, Brewer, Catmur, & Bird, 2017).

The renewed interest in interoception as a topic of scientific study has been driven by two complementary research aims. The first is concerned with establishing the extent to which interoceptive ability contributes to typical cognition, whereas the second assesses the clinical impact of atypical interoceptive ability. With respect to typical cognition, interoception has been shown to contribute toward various aspects of learning (Katkin, Wiens, & Ohman, 2001), decision making (Werner, Jung, Duschek, & Schandry, 2009), and emotional processing (Füstös, Gramann, Herbert, & Pollatos, 2013; Schandry, 1981; Terasawa, Fukushima, & Umeda, 2013; Wiens, Mezzacappa, & Katkin, 2000). This evidence of the role of interoception in typical cognition is consistent with clinically focused research that has highlighted the relevance of atypical interoception for mental health (Brewer, Cook, & Bird, 2016; Khalsa & Lapidus, 2016; Naqvi & Bechara, 2010; Paulus & Stein, 2006; Quattrocki & Friston, 2014; Verdejo-Garcia, Clark, & Dunn, 2012). Indeed, within psychiatry and clinical psychology, there is a relatively long theoretical history suggesting a causal role for atypical interoception across psychiatric and neurological disorders (Barrett & Simmons, 2015; Brewer, Cook, & Bird, 2016; Quattrocki & Friston, 2014), with long-standing claims of reduced awareness of interoceptive signals in feeding and eating disorders (Khalsa & Lapidus, 2016; Klabunde, Acheson, Boutelle, Matthews, & Kaye, 2013; Pollatos et al., 2008), hyperawareness of interoceptive signals in anxiety (Khalsa & Lapidus, 2016; Paulus & Stein, 2006), and panic disorder (Clark et al., 1997; Ehlers, 1993; Ehlers & Breuer, 1992).

Among contemporary theories of the contribution of interoceptive ability to psychopathology, one of the most well developed is that of Quattrocki and Friston (2014), which maps in impressive detail how an interoceptive impairment can cause the social, sensory, and self-representation symptoms of autism spectrum disorder (henceforth autism). Although generally endorsing this theory, Brewer, Happé, Cook, and Bird (2015) have argued for two modifications. The first is that interoceptive deficit does not result in autism but instead characterizes alexithymia (a subclinical condition characterized by difficulty identifying and describing one’s own emotions [Nemiah, Freyberger, & Sifneos, 1976]), which frequently co-occurs with autism (Berthoz & Hill, 2005). This suggestion has been tested empirically; in support of Brewer and colleagues’ contention that when autism and alexithymia are dissociated, it is alexithymia and not autism that is associated with interoceptive ability (Gaigg, Cornell, & Bird, 2016; Shah, Hall, Catmur, & Bird, 2016). The second suggested theoretical modification relates to the scope of impairment expected to result from atypical interoception. Whereas Quattrocki and Friston (2014) argued for a wide-ranging impact on socioemotional ability, including deficits in imitation, theory of mind, empathy, and emotion recognition, Brewer, Happé, Cook, and Bird (2015) argued that evidence suggests that ability in several of these domains dissociates (e.g., Happé, Cook, & Bird, 2017), making a single-factor explanation of competence across socioemotional domains unlikely.

The link between alexithymia and atypical interoception (Brewer, Cook, & Bird, 2016; Gaigg et al., 2016; Herbert, Herbert, & Pollatos, 2011; Longarzo et al., 2015; Shah et al., 2016) has prompted the claim that atypical interoception represents a core impairment across psychiatric disorders (Brewer, Cook, & Bird, 2016; Murphy et al., 2017). This claim is based on studies demonstrating the existence of the ‘p-factor’, a factor representing lesser to greater severity of psychopathology with associated disruption in neural circuitry, derived from factor analytic studies of symptom structure across diagnostic categories (Caspi et al., 2014; Laceulle, Vollebergh, & Ormel, 2015; Lahey et al., 2012). While earlier work described the existence of the p-factor, noting that individuals exhibiting high levels of symptom severity in one domain (e.g., alcohol dependence) were likely to experience severe symptoms in several other domains (e.g., obsessive–compulsive tendencies or anxiety), the cause of the intercorrelation between symptom severity across domains was unspecified. The hypothesis that it is interoceptive ability that drives symptom intercorrelation, and therefore that gives rise to the symptom co-occurrence evidenced by the p-factor, is consistent with the finding that interoception has been shown to affect some of the most fundamental cognitive processes including learning (Katkin et al., 2001), decision making (Werner et al., 2009), emotion processing (Füstös et al., 2013; Schandry, 1981; Terasawa et al., 2013; Wiens et al., 2000), and cognitive control (Sueyoshi, Sugimoto, Katayama, & Fukushima, 2014), all of which are likely to impact upon a range of symptoms. For example, poor interoception may result in atypical perception of reward and punishment, which in turn may cause atypical learning via operant conditioning, and impact on decision making. Furthermore, atypical interoception may result in aberrant perception of internal signals of one’s emotional state, resulting in delayed or less effective use of emotion regulation strategies.

The claim that interoception underlies the p-factor is also consistent with the fact that a large-scale meta-analysis of brain morphology across six distinct psychiatric disorders identified left and right insula and dorsal anterior cingulate, areas thought to subserve interoception (Craig, 2002; Critchley & Harrison, 2013; Critchley, Wiens, Rotshtein, Ohman, & Dolan, 2004; but see Damasio, Damasio, & Tranel, 2013; Feinstein et al., 2016) as the only areas of gray matter loss common to all disorders (Goodkind et al., 2015). However, the central piece of evidence for the claim that interoceptive ability gives rise to the p-factor is the increased prevalence of alexithymia across psychiatric disorders and evidence linking alexithymia with atypical interoception (Brewer, Cook, & Bird, 2016; Gaigg et al., 2016; Herbert et al., 2011; Longarzo et al., 2015; Shah et al., 2016). If alexithymia is a valid marker of atypical interoception, then the almost universally increased prevalence of alexithymia across psychiatric disorders is the strongest evidence yet for the idea that atypical interoception may underlie the symptom commonalities between disorders.

It should be noted, however, that although most interoceptive theories of mental health assume a unitary view of interoceptive ability (that interoceptive ability is stable, regardless of the particular interoceptive signal to be perceived), recent studies challenge this assumption. While the vast majority of work assessing interoceptive ability has used standard tests of cardiac perception (Schandry, 1981), an increasing number of studies have tested interoceptive ability in different interoceptive domains, partly because of concerns over the validity of cardiac tests (Khalsa, Rudrauf, Sandesara, Olshansky, & Tranel, 2009) but also to test the assumption of a unitary interoceptive ability. Although earlier studies supported a unitary view, reporting moderate correlations between tests of gastric and cardiac perception (Herbert, Muth, Pollatos, & Herbert, 2012; Whitehead & Drescher, 1980), recent work has reported a lack of correlation between other interoceptive domains (e.g., respiratory and cardiac; Ehlers & Breuer, 1992; Garfinkel, Manassei, et al., 2016; Pollatos, Herbert, Mai, & Kammer, 2016; Steptoe & Vögele, 1992). Furthermore, at the neural level, whilst the transmission of interoceptive information follows common pathways before its representation within insular and cingulate cortices, different receptors support the transduction of interoceptive signals across interoceptive domains (e.g., Craig, 2002). This neural architecture would therefore be consistent with both a unitary interoceptive ability and independent interoceptive abilities dependent on the signal to be perceived. If interoceptive ability does vary, then the validity of theories claiming a role for interoception in the etiology of clinical disorders (supported by an increased prevalence of alexithymia) needs to be assessed in interoceptive domains other than the perception of cardiac information. Furthermore, a fractionated interoceptive ability may have substantial implications for Brewer and colleagues’ (Brewer, Happé, Cook, & Bird, 2015) suggested modifications of Quattrocki and Friston’s (2014) interoceptive theory of autism. For example, it is possible that interoceptive ability in some domains may, after all, be associated with autistic symptom severity rather than alexithymia. In addition, although speculative, it is possible that if interoception is fractionated, then interoceptive ability may determine the full range of socioemotional ability suggested by Quattrocki and Friston (2014) and that dissociations are observed between different socioemotional abilities (Happé et al., 2017) because they rely on interoceptive ability in different domains. Thus, it is crucial to test whether interoceptive ability is associated with alexithymia across interoceptive domains.

Accordingly, this paper reports three experiments, each using a novel interoceptive test, which examine the association between alexithymic and autistic traits and individual differences in noncardiac interoception. Experiment 1 assesses individual propensity to use interoceptive information in the respiratory domain, whereas Experiments 2 and 3 assess the ability to form an accurate percept of interoceptive information in the domains of muscular effort and taste, respectively. If alexithymia is confirmed as a marker of interoceptive impairment, regardless of the nature of the interoceptive signal, then interoceptive theories of mental health will gain an important source of support. Furthermore, given the increased prevalence of alexithymia across psychiatric disorders, evidence linking alexithymia and poor interoception across interoceptive domains would make it likely that a number of psychiatric disorders are characterized by a multidomain failure of interoception. Conversely, if alexithymia is only associated with interoceptive ability in a limited range of interoceptive domains, then either interoceptive ability is unlikely to explain symptom intercorrelation across psychiatric disorders or the impact of atypical interoception across symptom domains is mediated by perception of a very restricted range of interoceptive signals. Finally, if autistic traits are associated with interoceptive ability, then crucial evidence for Quattrocki and Friston’s (2014) model of autism will have been provided—supporting one of the few theories of autism, which is able to address the condition from anatomical, genetic, computational, psychological, and behavioral perspectives.

## Experiment 1

**Figure 1 fig1:**
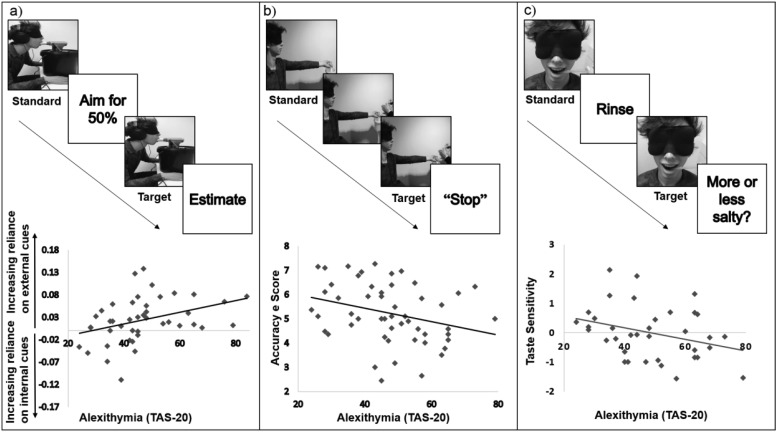
(a) Example trial in the respiratory task; participants were asked to estimate their ability to produce a target exhalation defined with respect to a standard exhalation under conditions manipulating reliance on external and internal cues. The difference in estimation accuracy between internal and external cue conditions is plotted, demonstrating alexithymia was associated with a reliance on external cues. (b) Example trial in the muscular effort task; participants indicated when the target weight matched that of the standard. Alexithymia was associated with reduced accuracy. (c) Example trial in the taste task; participants reported whether the target solution was more or less salty than the standard. Taste sensitivity was modeled by fitting psychometric functions, the plot demonstrates that increasing levels of alexithymia were associated with poorer taste sensitivity, even after controlling for a number of potentially confounding variables (see text for details).

Interoceptive ability is a multidimensional construct and can usefully be dissociated into the different dimensions of interoceptive accuracy and sensibility (Garfinkel & Critchley, 2013; Garfinkel, Seth, Barrett, Suzuki, & Critchley, 2015; McFarland, 1975; Terasawa et al., 2013; Whitehead, Drescher, Heiman, & Blackwell, 1977). Interoceptive accuracy is a measure of the degree to which one can accurately perceive the internal state of one’s body, whereas interoceptive sensibility reflects the propensity to become aware of interoceptive information and to be focused internally (Garfinkel et al., 2015). Interoceptive sensibility is thought to be reduced in alexithymia (Brewer, Cook, & Bird, 2016; Longarzo et al., 2015) and autism (Garfinkel, Tiley, et al., 2016); in common with interoceptive accuracy however, interoceptive sensibility can vary across interoceptive domains (Ehlers & Breuer, 1992). Experiment 1 therefore evaluated the impact of autistic and alexithymic traits on interoceptive sensibility in a noncardiac domain. Furthermore, previous studies have reported interoceptive sensibility to be, at least partly, independent from interoceptive accuracy (Chentsova-Dutton & Dzokoto, 2014; Garfinkel, Manassei, et al., 2016; Garfinkel, Tiley, et al., 2016; Khalsa et al., 2008). Importantly, these studies compare an objective, performance measure of interoceptive accuracy with a self-report measure of interoceptive sensibility. Although a perfectly valid approach, it remains ambiguous whether the lack of correspondence between the measures is a product of the dimension being tested (the accuracy of interoceptive perception vs. the propensity to become aware of interoceptive information) or the nature of the test (objective vs. subjective). The development of an objective test of interoceptive sensibility is therefore urgently required. Accordingly, Experiment 1 assessed interoceptive sensibility in the respiratory domain using a novel objective performance measure (see Figure 1a and *Methods*).[Fig-anchor fig1]

## Method

### Participants

Fifty-two participants took part in Experiment 1. Eight participants were excluded from the analysis owing to missing data, resulting in 44 usable data sets (*M*_age_ = 19.95, *SD*_age_ = 2.17, range 18–27, 13 males). Participants were selected on the basis that they had no known psychiatric or neurological conditions and had no history of breathing difficulties (e.g., asthma). Scores on the Autism Quotient (AQ; Baron-Cohen, Wheelwright, Skinner, Martin, & Clubley, 2001) were missing for one participant for which the mean score for all participants was entered. Eight participants met cutoff for alexithymia (Toronto Alexithymia Scale (TAS-20; Bagby, Parker, & Taylor, 1994); *M* = 47.07, *SD* = 14.17, range 24–84), and two participants met cutoff for autism (*M* = 17.02, *SD* = 8.06, range, 3–35). Alexithymic and autistic traits were not correlated in this sample, *r*(44) = .138, *p* > .05. Ethical clearance was granted by the local ethics committee. In line with the Declaration of Helsinki, all participants gave informed consent and were fully debriefed upon task completion. Each participant received either course credits or a small honorarium in exchange for participation. No significant differences in the main dependent variable (difference error scores; see *Results*) were found between paid (*M* = 0.21, *SD* = .048) and credit-receiving (*M* = 0.26, *SD* = .052) participants, *t*(42) = 0.32, *p* > .05, *d* = 0.098, 95% confidence interval (CI) for *d* [−0.027, 0.036].

### Materials

To quantify individual differences, two questionnaires were used: the TAS-20 (Bagby et al., 1994), a 20-item measure of alexithymic traits, and the AQ (Baron-Cohen et al., 2001), a 50-item measure of autistic traits. A standard peak flow meter (Wright, 1978) was used to gauge participants’ speed of exhalation (‘respiratory output’). The peak flow meter is a medical device that calculates the maximal peak of air flow in one exhalation (peak expiratory flow), measured in liters per minute. This gauge was gently secured in a horizontal position using a vice clamp and elevated in line with each participant’s mouth using a stand (see Figure 1a). For each participant disposable mouth pieces were used.

### Procedure

Upon arrival in the laboratory, participants completed the two questionnaires before the respiratory task. They were then given the following instructions: “In this experiment you will be asked to complete a large exhalation into the peak flow meter, a device that measures the maximum speed that you can push air out of your lungs. On each round this first exhalation will be taken as 100%. You will then be given an aim of 30%, 50%, 70%, 90% of that exhalation and asked to complete a second exhalation aiming for that percentage. After this you will be asked to estimate where you actually got to as a percentage of the first exhalation.” The instructions were followed by a demonstration from the experimenter using a mouthpiece that was not attached to the gauge.

On each trial, participants were required to perform a large exhalation into the peak flow meter, on the experimenter’s count of three. This first exhalation was taken as their standard (100%) for that trial and was noted by the experimenter. They were then given a target (e.g., 50% of their first exhalation) and asked to perform a second exhalation on the experimenter’s count of three. The value recorded by the peak flow meter for their second exhalation was noted by the experimenter. Following this target exhalation, they were asked to estimate their performance as a percentage of the standard, and this value was recorded. This procedure was completed under two conditions, internal and external. In the internal condition, each exhalation was accompanied by white noise played through headphones (Philips SHP2000 Over-Ear Corded Audio Headphones Amsterdam, the Netherlands) connected to a laptop (Asus Zenbook ux305 Taipei, Taiwan) for 4 seconds (∼79 decibels) so that auditory information relating to the exhalation was not available to aid performance. The white noise was started by the experimenter on the count of two. In the external condition, each exhalation was accompanied by 4 seconds of white noise played externally through the laptop speakers (∼79 decibels), starting on the count of two, with the laptop placed approximately 1 meter away from the right side of the participant. Auditory information relating to respiratory output was therefore available for use on the external condition, whilst the distracting effect of the white noise was approximately equated across conditions. In both conditions participants were blindfolded to prevent them from using the values on the gauge to estimate their performance. The order of these conditions was fully counterbalanced across participants. In each condition participants completed six blocks of four trial targets (30%, 50%, 70%, 90%), with the order of targets randomized across each block.

To ensure that the smallest target (30%) could be measured, participants were required to blow over a threshold that was set at 200 L/min on their standard exhalation. If the participant’s standard exhalation fell below this threshold, it was repeated until above-threshold performance was reached. On rare occasions where exhalations fell between two values on the gauge, the experimenter always rounded up to the nearest value. Prior to the experiment, one practice trial with a target of 50% was performed to ensure participants could comfortably reach the threshold and understood the task instructions. No feedback was provided at any point during the experiment. Prior to the experimental block with headphones, participants were also given an example of the white noise to ensure it was at a comfortable level. Participants were informed that their hands must rest either at the bottom of the stand or on the table during exhalations. They were permitted to use only their hands to locate the mouthpiece between trials before replacing them prior to exhalation. Participants were also asked to sit upright in the chair and not push forward onto the mouthpiece during exhalations. Trials in which participants failed to follow these instructions were either repeated where possible or removed from the analysis.

## Results

### Data Analysis

For each trial (for both external and internal conditions) absolute error scores (absolute [(actual second exhalation as a percentage of the standard—participant’s estimate)/actual second exhalation as a percentage of the standard]) were computed (e.g., if the standard exhalation was 500 and the second exhalation 250, then the actual second exhalation as a percentage of the standard would be 50%. If the participant estimated 40%, the equation would be as follows: absolute [(50 – 40)/50], resulting in an error score of 0.2 for that trial). For each participant, in each condition, mean error scores were calculated. From these mean values, difference error scores (absolute error internal – absolute error external) were derived, quantifying the difference between performance on the internal and external conditions. Negative values represent better performance (less error) on the internal condition, values around zero indicate performance was not aided by the addition of exteroceptive information in the external condition, and positive values indicate better performance on the external condition. Trials for which targets were beyond the range of the peak flow meter (<60 L/min), or the participant failed to follow instructions, were excluded from the analysis. Participants missing more than 8% of trials in any one condition were removed from further analysis. Two researchers collected data for this experiment, interrater reliability tests (to ensure consistent rounding up of values that fell between 2 points on the gauge) confirmed good reliability, *K* = .500, *p* < .0005, (95% CI [0.447, 0.653]), and difference error scores did not significantly vary between researchers 1 (*M* = 0.06, *SD* = 0.05) and 2 (*M* = 0.02, *SD* = 0.05), *t*(42) = .319, *p* > .05, *d* = 0.124, CI for *d* [−0.485, 0.730]. The Kolmogorov-Smirnov test was used to assess normality and indicated the data were normally distributed (*D* = .113, *p* > .05). In addition, to quantify participants’ ability to control respiratory output, average respiratory control scores were calculated according to the formula absolute [(target percentage – actual percentage exhaled)], and controlled for in the analysis to ensure that participants’ ability to perceive the internal state of their body (interoception) was not influenced by their ability to control their respiratory output.

The difference in estimation accuracy between internal and external conditions served as a performance measure of interoceptive sensibility and was associated with alexithymic traits, *r*(44) = .354, *p* = .018, such that increased alexithymia was associated with a reduced reliance on interoceptive information. This association remained after controlling for both autistic traits and ability to control respiratory output, *r*(40) = .321, *p* = .038. Neither autistic traits, *r*(44) = .017, *p* > .250 nor alexithymia was associated with the ability to control respiratory output, *r*(44) = −.173, *p* > .250. Participants reporting lower levels of alexithymia exhibited no performance benefits with the addition of exteroceptive information (median split: low alexithymia group difference scores compared against zero, *t*[20] = .084, *p* > .250, *d* = 0.02, 95% CI for *d* [−0.410, 0.446]), indicating a complete reliance on interoceptive information, whereas those higher in alexithymic traits performed better with the addition of exteroceptive information (high alexithymia group *t*([22] = 5.51, *p* < .001, *d* = 1.15, 95% CI for *d* [0.611, 1.671]). Using the same measure, interoceptive propensity was not associated with autistic traits, *r*(44) = .224, *p* > .05. Results therefore support the characterization of alexithymia as a general interoceptive impairment (Brewer, Cook, & Bird, 2016) but question whether autism is associated with a reduced propensity to utilize interoceptive information.

### Experiment 2

Experiment 1 used a performance measure to assess the degree to which alexithymic and autistic traits were associated with interoceptive sensibility in a noncardiac domain. Experiment 2 instead assessed interoceptive accuracy, the degree to which participants can form an accurate percept of their body’s internal state, in an additional noncardiac interoceptive domain: muscular effort (Wilson et al., 2002).

### Participants

Fifty-two participants (*M*_age_ = 20.02, *SD*_age_, 2.93, range 18–32, 12 males, four left handed) took part in Experiment 2. Participants were selected on the basis that they had no known psychiatric or neurological conditions and had no history of shoulder, wrist, or arm injuries. Ethical clearance was granted by the local ethics committee. In line with the Declaration of Helsinki, all participants gave informed consent and were fully debriefed upon task completion. All participants received either course credits or a small honorarium in exchange for participation. No difference in interoceptive accuracy (accuracy scores; see *Results*) was found between paid or credit-receiving participants, *t*(50) = 1.057, *p* > .05, *d* = 0.304, 95% CI for *d* [−0.265, 0.871]. AQ scores were missing for one participant for whom the mean score for all participants was entered. Nine participants met the cutoff for alexithymia (*M* = 47.29, *SD* = 13.08, range 24–79) and six for autistic traits indicative of autism (*M* = 19.79, *SD* = 9.67, range, 3–45). In this sample there was a trend for high rates of alexithymia to be associated with higher autistic traits, *r*(52) = .241, *p* = .085.

### Materials

For Experiment 2 three identical 1-L buckets were filled with rice so that the total weight of each sealed bucket was 350, 510, or 780 g. These amounts were randomly selected but chosen to be an integer multiple of ten.

### Procedure

As in Experiment 1, participants initially completed two questionnaires: the TAS-20 (Bagby et al., 1994) and the AQ (Baron-Cohen et al., 2001). All participants were then given the following instructions:

On each round a bucket filled with rice will be placed onto the upright palm of your dominant hand for 2 seconds. You will be asked to gently close your fingers when holding the bucket. It will then be replaced by an empty bucket, which the experimenter will fill with rice at a constant speed. You will be asked to say stop when you think that the bucket weighs the same as the previous bucket you were holding. It is very important that you keep your arm completely straight and at a 90-degree angle throughout the task. You should say stop only when you are absolutely certain the bucket weighs the same. Do not say stop because you are worried the bucket will overflow or that it has been pouring for a long time.

Participants were then given an example of this procedure with an empty bucket as the standard that was not filled with rice to ensure correct hand and arm positioning. The experimenter always placed the handle of the bucket over the metacarpophalangeal joint and ensured the participant’s arm was straight in front of them, in line with their shoulder, with their palm facing upward (Figure 1b). Participants were blindfolded throughout the task to ensure they could not use visual cues to gauge the weight of the bucket. After each trial the bucket was weighed by the experimenter and the weight was noted. Each participant completed one trial with each of the three buckets, the order of which was fully counterbalanced across participants. The task therefore required the participant to be able accurately to perceive the muscular effort required to hold the standard and target weights in an isometric position and to determine when these signals matched.

## Results

### Data Analysis

As in Experiment 1, absolute error scores were computed (absolute [(actual weight of the standard − participant’s estimate of the weight of the target bucket)/actual weight of the standard]) for each trial, which were then averaged. The Kolmogorov-Smirnov test was used to assess normality and indicated marginal negative skew (*D* = .125, *p* = .040). To correct the data, a square root transformation was performed. As a result, high scores indicate good performance, whereas low scores represent increased error.

Interoceptive accuracy (the absolute difference between the standard and target weights and thus the participants’ ability to detect when the standard and target weights matched) was associated with alexithymic traits, *r*(52) = −.296, *p* = .033, even after controlling for autistic traits, *r*(49) = −.335; *p* = .016, such that an increasing degree of alexithymic traits was associated with poorer interoceptive accuracy. In contrast, interoceptive accuracy was not associated with autistic traits, *r*(52) = .111, *p* > .05.

### Experiment 3

Experiment 3 provided another test of the association between interoceptive accuracy and alexithymic and autistic traits with three novel features. First, interoceptive accuracy was assessed in a novel domain: taste (Craig, 2004; Critchley & Harrison, 2013). Second, an exteroceptive control task was included to ensure that any association with interoceptive accuracy was specific to the perception of interoceptive signals, rather than due to general effects such as attention, motivation, or differences in working memory. Third, both alexithymia and autism are associated with increased rates of depression and anxiety (Marchesi, Brusamonti, & Maggini, 2000; Strang et al., 2012); therefore, the effect of these traits was controlled for in Experiment 3.

## Method

### Participants

Thirty-eight participants completed Experiment 3. One participant was removed owing to a failure to follow instructions, and one outlier was removed resulting in 36 usable cases [*M*_age_ = 21.03, *SD*_age_ = 3.44, range 18–34, five males]. Control task data were missing for one participant for whom the mean score was entered. All participants were selected on the basis that they had no known psychiatric or neurological conditions and had English as their first language or a high level of English proficiency. Ethical clearance was granted by the local ethics committee. In line with the Declaration of Helsinki, all participants gave informed consent and were fully debriefed upon task completion. All participants received either course credits or a small honorarium in exchange for participation. Only three participants received credits, but no significant differences were observed between paid and credit-receiving participants on the taste task, *t*(34) = .524, *p* > .05, *d* = 0.316, 95% CI for *d* [–.870, 1.498] or the exteroceptive control task, *t*(34) = 1.349, *p* > .05, *d* = 0.813, 95% CI for *d* [–.390, 2.005].

Eleven participants met the cutoff for alexithymia (*M* = 48.47, *SD* = 14.41, range 24–79) and two for autistic traits indicative of autism (*M* = 17.00, *SD* = 8.93, range 3–36). A typical range of scores was observed for state anxiety (*M* = 32.61, *SD* = 8.83, range 20–60), trait anxiety (*M* = 41.17, *SD* = 9.49, range 23–61), and depression (*M* = 8.39, *SD* = 6.77, range 0–27). In this sample alexithymia was positively associated with autistic traits, *r*(36) = .359, *p* = .031, depression, *r*(36) = .459, *p* = .005, and trait anxiety, *r*(36) = .536, *p* < .001. There was also a trend for alexithymia to be associated with higher state anxiety, *r*(36) = .318, *p* = .066. Likewise, autistic traits were positively associated with trait anxiety, *r*(36) = .598, *p* < .001, and state anxiety, *r*(36) = .461, *p* = .005. There was also a trend for autistic traits to be associated with higher depression scores, *r*(36) = .313, *p* = .063.

### Materials

Four questionnaires were used, the TAS-20 (Bagby et al., 1994), the AQ (Baron-Cohen et al., 2001), the State and Trait Anxiety Inventory (Spielberger, Gorsuch, Lushene, Vagg, & Jacobs, 1983), and the Beck Depression Scale (Beck, Steer, & Brown, 1996).

### Taste Task

For the taste task, seven solutions of salt water were created, ranging from .102–.292 mol in steps of 16%. This stepwise selection was determined by extensive piloting and informed by prior research into the just noticeable difference for taste solutions (Schutz & Pilgrim, 1957). These solutions were made using 99.9% pure NaCl (Sigma-Aldrich, St. Louis, MO) and distilled water (www.distilledwatercompany.com). All solutions were made a maximum of 2 weeks prior to the experiment and stored in sealed containers at a constant temperature of 21°C away from direct sunlight. For each participant seven plastic disposable pipettes, one for each solution, were used.

The participant was presented with the standard (.197 mol) at the beginning of each trial. Participants were then presented with a target solution from one of the seven stimulus levels. Across the experiment, 16 blocks of seven trials were completed, one trial per block for each of the seven levels. The order in which the targets were presented was randomized across each block.

### Exteroceptive Control Task

The exteroceptive control task format was identical in format to the taste task and was created in Matlab 8.0 (Mathworks, Natick, MA) with the Cogent 2000 toolbox (http://www.vislab.ucl.ac.uk/Cogent). Stimuli were presented on a Toshiba Satellite laptop computer, with a 60-Hz refresh rate and screen size of 15.6 inches. Seven gray patches (495 × 428 pixels) were created, with the fourth patch taken as the standard. The six remaining patches ranged from −30% to +30% on either side of the standard in red, green, and blue color change steps of 10%. This selection was determined through extensive piloting. On each trial the standard color patch was presented for 1000 ms in the center of the screen. Following an interstimulus interval of 1000 ms, one of the seven target patches was presented for 1000 ms in the center of the screen. Following stimulus offset, the user was prompted to select whether the target was “brighter or darker” than the standard by pressing the left or right arrow key, respectively. This response immediately triggered the start of the next trial. Sixteen blocks of each of the seven targets were used and target order was fully randomized across each block.

### Procedure

Following questionnaire completion, participants completed the taste and exteroceptive control tasks, the order of which was counterbalanced across participants.

### Taste Task

Prior to the taste task, participants were asked to rinse and gargle with distilled water. On each trial, participants were given 2 ml of the standard taste solution that was pipetted under their tongue using a disposable pipette (Figure 1c). They were asked to taste the solution and then spit it into a bucket. This was followed by a rinse and spit with distilled water. After rinsing, participants were given 2 ml of a target solution, pipetted in the same way as the standard. After spitting this out, they were asked to state whether the second solution was more or less salty than the first. This was followed by a second rinse and spit with distilled water prior to the next trial. During the task the experimenter always said “number one” when presenting the standard and “number two” when presenting the target. This was always followed by the same prompt: “Was the second one more or less salty?” Participants were given one practice trial prior to the experiment during which the standard was presented twice. No feedback was provided. All participants were allowed a break halfway through the experiment. Throughout the experiment participants were blindfolded to ensure they could not learn to associate a particular solution with a certain intensity.

### Exteroceptive Control Task

During the exteroceptive control task, participants were seated approximately 60 cm away from the computer screen in a dimly lit room. The following instructions were presented: “In this experiment you will see two gray squares. Your task is to decide whether the second square is brighter or darker than the first. Press left for brighter (<) and right for darker (>). When you are ready, please press space to begin the practice.” Participants were given one practice block to familiarize themselves with the response keys and a break was given halfway through the experiment.

## Results

### Data Analysis

Both the taste task and the exteroceptive control task were analyzed by fitting psychometric functions to participants’ judgments of whether the stimulus was greater than (e.g., more salty or brighter) or less than (e.g., less salty or dimmer) the standard. Thus, both tasks required the participant to identify the direction of stimulus discrepancy between the target and the standard. Separate cumulative Gaussian functions were fitted for each participant based on 112 observations (16 presentations × 7 stimulus levels) using the Palamedes toolbox (Prins & Kingdom, 2009). Each function estimated one parameter of interest, the slope. Slope estimates measure the precision with which stimuli are categorized; Steep and shallow slopes are associated with low and high noise estimates, respectively. Low-slope estimates indicate insensitivity to stimulus strength and therefore inaccurate categorization. Therefore, participants’ taste and color sensitivity (and therefore interoceptive/exteroceptive accuracy) was indexed by the slope of their psychometric function, with steeper slopes indicating more precise categorization. Slope measures were free to vary and estimated initially at 50% and 10%, respectively. Guess and lapse rates were fixed at 0.

Analysis of the interoceptive data was carried out using hierarchical regression. Participant age, gender, depression, state, and trait anxiety scores were entered into the first step of the regression model, autistic traits into the second, and alexithymia into the third. Exteroceptive sensitivity scores were also entered into the first step so that any variance accounted for by alexithymia or autistic traits was specific to the interoceptive task and not because of nonspecific factors such as motivation, working memory, or other general cognitive factors. At Step 1, only state anxiety predicted worse taste sensitivity (standardized β = −.787, *t* = −3.509, *p* < .001, 95% CI for β [−0.468, −0.123], Δ*R*^2^ = 23.7%). All other predictors were nonsignificant (all β < .516; all *p* > .05). The overall model was significant, *F*(6, 29) = 2.814, *p* = .028. When autistic traits were added (Step two), only state anxiety (β = −.785, *t* = −3.445, *p* = .002, 95% CI for β [−0.471, −0.120]) predicted worse taste sensitivity. The inclusion of autistic traits did not increase the variance accounted for by the model, 0.1%, *F*(1, 28) = .066, *p* > .05, and the overall regression model was not significant, *F*(7, 28) = 2.343, *p* = .051. When alexithymia was added (Step 3), both state anxiety (β = −.843, *t* = −3.958, *p* < .001, 95% CI for β [−0.481, −0.153]) and alexithymia (β = −.422, *t* = −2.343 *p* = .027, 95% CI for β [−0.182, −0.012]) predicted worse taste sensitivity. There was also a trend for trait anxiety to predict better taste sensitivity (β = .690, *t* = 1.987, *p* = .058, 95% CI for β [−0.009, 0.492]). The inclusion of alexithymia significantly increased the variance accounted for by the model, 10.7% *F*(1, 27) = 5.489, *p* = .027, and the overall model was significant, *F*(8, 27) = 3.066, *p* = .014.

The equivalent analysis was completed on data from the exteroceptive control task. Participant age, gender, depression, taste sensitivity scores, state, and trait anxiety scores were entered into the first step of the regression model, autistic traits into the second, and alexithymia into the third. At all steps none of the variables predicted exteroceptive sensitivity (all *p* > .20) and the overall model was not significant *F*(8, 27) = .879, *p* > .50). However, although the residuals were normally distributed (*D* = .115, *p* > .05), the relationship between the predicted and observed residuals was not normal. To further confirm the absence of a relationship between alexithymia and the exteroceptive control task, a Spearman’s rank-order correlation was conducted, which confirmed that performance on the exteroceptive control task did not correlate with alexithymia, *r*(36) = .180, *p* > .250.

## General Discussion

This set of three studies aimed to assess claims of a link between alexithymia and impaired interoception in the light of recent evidence that interoceptive ability, presently assessed almost exclusively within the cardiac domain, may vary depending on the interoceptive signal to be perceived. Experiment 1 utilized a novel measure of interoceptive sensibility in the respiratory domain to reveal that individuals high in alexithymic traits (but not autistic traits) relied on exteroceptive information when judging respiratory output, whereas those low in alexithymic traits relied on interoceptive information. Experiments 2 and 3 assessed interoceptive accuracy in two novel domains: muscular effort and taste. In each case increasing alexithymic traits (but not autistic traits) were associated with less accurate perception of interoceptive information. Furthermore, Experiment 3 established that the relationship between alexithymic traits and interoceptive accuracy was specific to interoception—there was no relationship between alexithymic traits and a closely matched exteroceptive control task—and not an artifact of co-occurring depression or anxiety.

The current results are consistent with the proposal that alexithymia may be a marker of a multidimensional, multidomain, interoceptive impairment—associated both with reduced interoceptive accuracy and decreased integration of interoceptive information with ongoing cognition regardless of the interoceptive signal under consideration. Indeed, given discrepancies between self-reported interoceptive awareness and interoceptive accuracy (e.g., Garfinkel et al., 2015), these data are consistent with existing data suggesting that self-reported alexithymia may be a useful screening tool for identifying those with poor interoception (Brewer, Cook, & Bird, 2016; Gaigg, Cornell, & Bird, 2016; Herbert et al., 2011; Longarzo et al., 2015; Shah et al., 2016).

This evidence that alexithymia may be a marker of atypical interoception is in line with the proposal by Brewer and colleagues (Brewer, Cook, & Bird, 2016) that interoceptive ability may underlie the existence of the p-factor, a first-order overarching factor representing lesser to greater severity of psychopathology and associated neural dysfunction identified by confirmatory factor analytic work on symptom co-occurrence across diagnostic categories (Caspi et al., 2014; Laceulle et al., 2015; Lahey et al., 2012). Indeed, the link between interoception and alexithymia, together with evidence linking alexithymia and various psychiatric disorders, raises the possibility that atypical interoception may characterize several psychiatric conditions. As many symptoms contributing to the p-factor model (symptoms found across a range of disorders) may be driven by atypical interoception (e.g., addictive behaviors, weight change; for a detailed discussion of the mechanism by which interoception may contribute to addiction, see Verdejo-Garcia et al., 2012) or be inherently interoceptive (e.g., fatigue, muscle tension), this raises the possibility that the statistically observed p-factor is driven by a common deficit in interoception, which in turn impacts on a range of symptoms, accounting for observed symptom intercorrelations (see Murphy et al., 2017).

The lack of an association between autistic traits and interoceptive ability is not consistent with previous claims that autism is associated with interoceptive impairment (e.g., Quattrocki & Friston, 2014), although it should be noted that the current study did not assess interoception in individuals diagnosed with autism, and the association between autistic traits and interoception in the typical population may not be as strong as when tested in diagnosed individuals.

Although these findings are in line with previous studies reporting an association between alexithymia and poor interoception (Brewer, Cook, & Bird, 2016; Gaigg et al., 2016; Herbert et al., 2011; Longarzo et al., 2015; Shah et al., 2016), they may be considered surprising, given recent evidence that interoceptive ability depends upon the interoceptive signal to be perceived (Garfinkel, Manassei, et al., 2016; Pollatos et al., 2016; Steptoe & Vögele, 1992). If alexithymia is associated with interoceptive accuracy across cardiac, muscular, and taste interoceptive domains and with interoceptive sensibility across an even greater number of domains (Brewer, Cook, & Bird, 2016; Longarzo et al., 2015), then although not explicitly examined by this series of experiments, this is at least consistent with a unitary interoceptive ability, regardless of the interoceptive signal to be perceived. While any explanation of this paradox is necessarily speculative, it is of note that there has been little opportunity, given the interoceptive tasks that currently exist, to equate task demands across interoceptive domains. For example, some tasks measure participants’ ability to detect interoceptive stimuli (e.g., when an obstruction is applied to respiration [Garfinkel, Manassei, et al., 2016; Pollatos et al., 2016]); others assess the ability to determine the magnitude of interoceptive signals (such as in the muscular effort and taste tasks used in Experiments 2 and 3), whereas others measure the ability to discriminate between interoceptive signals (Giguère et al., 2016). Furthermore, tasks are not generally equated for speed, accuracy, working memory, or sustained attention demands across tasks; for example, the standard heartbeat tracking task (Schandry, 1981) requires a sustained period of attention for up to 100 seconds, whereas the respiratory task used by Pollatos and colleagues (Pollatos et al., 2016) required participants to sustain attention for only a few seconds during a period of exhalation. Finally, the degree to which interoception contributes to performance on several interoceptive tasks is not always clear because of the lack of matched exteroceptive control tasks. For example, the heartbeat tracking task is sometimes accompanied by a control task in which individuals are required to estimate the duration (in seconds) of time periods equivalent to those over which they count their heartbeats (Ainley, Brass, & Tsakiris, 2014; Shah et al., 2016). Although the duration estimation task has the same counting and sustained attention demands as the cardiac tracking task, participants are not required to detect exteroceptive signals (which could be matched to the average detectability of heartbeats), and therefore, the duration estimation task is not a fully matched control task: for example, it does not control for any response bias, which may affect performance on the cardiac tracking task. There is clearly an urgent need for further tests of interoceptive ability, with appropriate control tasks, that can be matched for difficulty, in order to address the question of whether interoceptive ability is invariant across interoceptive domains or whether it varies, depending on the signal to be perceived, across both typical samples and clinical populations.

**Figure 2 fig2:**
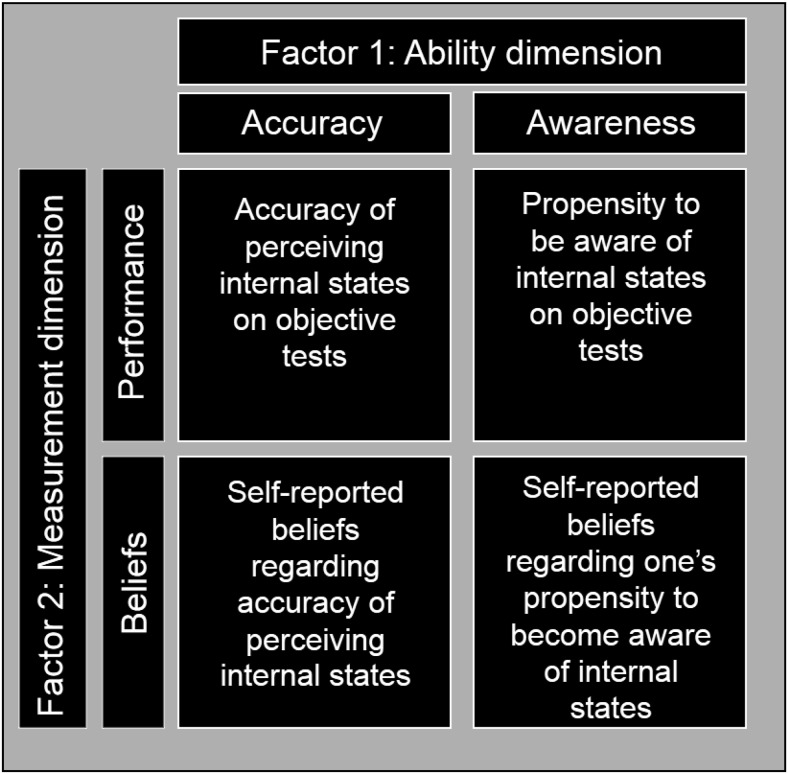
Illustration of the proposed 2 × 2 factorial structure of interoception. Factor 1 distinguishes between interoceptive accuracy (the ability to accurately perceive the internal state of one’s body) and awareness (the propensity to become aware of interoceptive signals). Factor 2 distinguishes between an individual’s beliefs in their interoceptive ability (self-report) and their objective performance on tests of interoception across Factor 1 dimensions (see text for details). Therefore, this model suggests four possible dimensions of interoception: (a) the ability to accurately perceive the internal state of one’s body as measured by objective tests (e.g., the heartbeat tracking task; Schandry, 1981). (b) the ability to accurately self-report one’s ability to perceive the internal state of one’s body; (c) one’s self-reported propensity to become aware of interoceptive signals (e.g., the Body Perception Questionnaire; Porges, 1993); and (d) one’s propensity to utilize internal signals as measured by objective tests (e.g., the respiratory output task; Experiment 1).

Experiment 1 reported a new test described as a measure of interceptive sensibility. In this task, performance was measured under two conditions that varied the availability of interoceptive and exteroceptive cues. Participants reporting lower levels of alexithymia exhibited no performance decrements when the availability of exteroceptive cues was curtailed, indicating this information was ignored when judging respiratory output, whereas those higher in alexithymic traits performed better with the addition of exteroceptive information. Previously, interoceptive sensibility has been measured via self-report and defined as “the individual’s belief in their interoceptive ability and the degree to which they feel engaged by interoceptive signals” (Garfinkel et al., 2015, p. 66). Although differentiation of interoceptive sensibility and accuracy has been hugely beneficial for the field, the results obtained in Experiment 1 suggest that it may be beneficial to further subdivide interoceptive sensibility, distinguishing between (a) self-reported interoceptive accuracy and (b) the awareness of interoceptive signals. We suggest a 2 × 2 factorial structure of interoception (illustrated in Figure 2) in which the first factor distinguishes between interoceptive accuracy and the propensity to become aware of interoceptive information (e.g., an individual may be typically unaware of interoceptive signals but perform well when explicitly asked to attend to interoceptive information. In such a case, the individual would have good interoceptive accuracy but a low propensity to become aware of interoceptive information). The second factor distinguishes one’s objective performance in each of these domains from one’s belief about the degree to which one can form accurate percepts of interoceptive states and one’s propensity to become aware of interoceptive information (e.g., distinguishing between an individual’s objective performance on tests of interoceptive accuracy and propensity from their self-reported beliefs regarding these dimensions of interoception). Under this account, the test described in Experiment 1 could be described as an objective measure of the propensity to be aware of interoceptive information; performance in a condition in which there is no requirement to rely on interoceptive information can be compared to conditions in which there is a requirement to depend on interoceptive information to determine the reliance on interoceptive information. It should be noted, however, that even this more fine-grained 2 × 2 structure is an oversimplification. One could also distinguish between the degree to which one is aware of interoceptive information and the degree to which one uses this information in tasks such as that used in Experiment 1. Furthermore, the use of interoceptive information is likely governed by the confidence one has in one’s own interoceptive accuracy; for example, individuals with greater confidence in their abilities may be more likely to utilize interoceptive signals.[Fig-anchor fig2]

In summary, these experiments confirm that alexithymia affects multiple dimensions and domains of interoception, consistent with proposals that atypical interoception may represent a common factor across psychopathology. This evidence also emphasizes the need for further subdivision of interoceptive sensibility, separating objective and subjective propensity to prioritize interoceptive signals, and highlights the need for further examination of interoceptive accuracy and how it varies across interoceptive domains using measures that are equated for task demands.

### Context Paragraph

A growing body of evidence indicates that alexithymia may best be considered a failure of interoception. Yet recent evidence that interoception may fractionate depending on the bodily signal to be perceived highlights the need to reassess these claims across unexamined domains of interoception. The aim of the authors was to build on existing research and extend this by examining whether alexithymia is associated with reduced accuracy and propensity to utilize interoceptive signals, which is related to their current work examining individual differences in interoception and the relationship between interoception and mental health.
